# Visual Acuity of Simulated Thalamic Visual Prostheses in Normally Sighted Humans

**DOI:** 10.1371/journal.pone.0073592

**Published:** 2013-09-27

**Authors:** Béchir Bourkiza, Milena Vurro, Ailsa Jeffries, John S. Pezaris

**Affiliations:** Department of Neurosurgery, Massachusetts General Hospital, Harvard Medical School, Boston, Massachusetts, United States of America; CSIC-Univ Miguel Hernandez, Spain

## Abstract

Simulation in normally sighted individuals is a crucial tool to evaluate the performance of potential visual prosthesis designs prior to human implantation of a device. Here, we investigated the effects of electrode count on visual acuity, learning rate and response time in 16 normally sighted subjects using a simulated thalamic visual prosthesis, providing the first performance reports for thalamic designs. A new letter recognition paradigm using a multiple-optotype two-alternative forced choice task was adapted from the Snellen eye chart, and specifically devised to be readily communicated to both human and non-human primate subjects. Validation of the method against a standard Snellen acuity test in 21 human subjects showed no significant differences between the two tests. The novel task was then used to address three questions about simulations of the center-weighted phosphene patterns typical of thalamic designs: What are the expected Snellen acuities for devices with varying numbers of contacts, do subjects display rapid adaptation to the new visual modality, and can response time in the task provide clues to the mechanisms of perception in low-resolution artificial vision? Population performance (hit rate) was significantly above chance when viewing Snellen 20/200 optotypes (Log MAR 1.0) with 370 phosphenes in the central 10 degrees of vision, ranging to Snellen 20/800 (Log MAR 1.6) with 25 central phosphenes. Furthermore, subjects demonstrated learning within the 1–2 hours of task experience indicating the potential for an effective rehabilitation and possibly better visual performance after a longer period of training. Response time differences suggest that direct letter perception occurred when hit rate was above 75%, whereas a slower strategy like feature-based pattern matching was used in conditions of lower relative resolution. As pattern matching can substantially boost effective acuity, these results suggest post-implant therapy should specifically address feature detection skills.

## Introduction

In the last few decades, millions of people facing acquired blindness have been offered reason for hope by the discovery that electric stimulation of the visual pathway can be used to generate artificial visual percepts, or *phosphenes* [1]. Recent work has demonstrated the feasibility of visual prosthesis devices able to transform images from a video camera into temporal patterns of electrical stimulation, and thus modulating patterns of phosphenes, that are perceived as rudimentary images by implanted subjects [2], [3], [4]. Despite tremendous advances, current state-of-the-art prosthetic vision remains somewhat crude, with substantial effort still required to produce high-quality artificial sight [5], [6].

Central to contemporary visual prosthesis approaches is the idea that passing current through single small-contact electrodes can be used to generate small, point-like phosphenes, and that collections of such electrodes can be used to manipulate sets of phosphenes like sets of pixels in a computerized display. However, unlike the pixels in a display, such phosphenes are not anticipated to form a continuous, dense surface in the visual field, but, rather, to be spatially isolated one from the next, therefore creating a pointillistic pattern. The precise spatial arrangement of phosphenes, driven by the precise anatomical placement of electrodes, is thought to be important in determining the utility of a visual prosthesis.

Given the large effort required to implant electrodes even in an animal model, and the difficulty with changing electrode positions once implanted, many research groups have turned to virtual reality techniques in the development of visual prostheses (*e.g.* [7], [8], 9,10,11,12,13). Published reports have concentrated on simulations of devices that interface with different stages of the visual pathway including the retina, the optic nerve, or the visual cortex, with those coupled to the lateral geniculate nucleus (LGN) of the thalamus, a mid-brain structure considered a relay station between retina and primary visual cortex [14], left almost unexplored [15], [16], [17].

Our group has detailed the motivations for studying thalamic prosthetic vision in previous reports, as summarized here. Pezaris and Reid [15] demonstrated the efficacy of thalamic microstimulation in generating phosphenes that were readily integrated into a visual task. Pezaris and Reid [18] analyzed the engineering and surgical aspects of multiple microwire implants in LGN to address practical issues. Pezaris and Eskandar [5] compared the different major areas for potential stimulation with the conclusion that the thalamic approach carries the promise of a large number of benefits, including the applicability to a wide range of causes of blindness, from retinal degeneration to glaucoma. Among the important aspects of the LGN approach were found to be foveal magnification, which creates the potential for high-resolution artificial vision, intra-cranial location, which provides a stable mechanical platform, and routine surgical access due to advances in the unrelated field of deep brain stimulation (reviewed in [19]), which allows safe implantation of stimulating electrodes in the midbrain. As the pattern of phosphenes in a thalamic prosthesis is anticipated to centrally-weighted [5], [18], the simulation literature that heretofore concentrated on the regular phosphene patterns from retinal designs was found to be only indirectly informative to thalamic designs, underscoring the need for additional investigation.

In the present report, we continued our line of inquiry into LGN-based prostheses by using virtual reality simulations of thalamic prosthetic vision to evaluate the performance of potential designs. We determined the acuity the designs would provide and therefore were able to bound the anticipated complexity required to broach the thresholds of legal blindness. The four goals of this study were to: (1) determine the effective acuity of thalamic implants with modest electrode count; (2) determine if subjects rapidly learn to utilize the new visual modality such devices represent; (3) determine if there are different subject strategies, or modes of prosthesis use, at different difficulty levels; and (4) introduce a novel computer-based visual acuity assessment equivalent to the familiar Snellen chart test.

The task created for this report was designed to be simple, easily communicated to both human and non-human primate subjects, and fully automatable, lending itself to future cross-species and cross-laboratory comparisons. The Snellen test was chosen as a basis for the paradigm because it is widely recognized, is a good predictor of many visually-guided task in daily activities [20], and provides a ready means of comparison with work from other groups. Combining the Snellen optotypes with a straightforward behavioral task, we created a multiple-optotype two-alternative forced-choice paradigm that is shown below to be an accurate tool for measuring visual acuity.

Within the task is a period of simulated prosthetic vision that includes gaze contingency in the generation of visual stimuli. This is an important aspect to the work and reflects the expectation that thalamic phosphenes are perceived relative to the instantaneous direction of gaze [15], in what is known as a *retinotopic reference frame* [21]. This feature of the simulation, which required substantial effort during software development, allows the dynamic generation of simulated phosphenes on a computer monitor that are approximately stabilized on the retina (Section 2.4 Simulating Thalamic Prosthetic Vision).

The novel task was used to conduct two experiments in normal, sighted human volunteers, a *Primary* experiment and a *Validation* experiment. In the Primary experiment, the responses of subjects to the letter recognition task were used to address the first three goals listed above that concern visual performance. In the Validation experiment, the task was used without simulated prosthetic vision to address the fourth goal, evaluating the novel paradigm by comparing it to the Snellen chart test commonly used by clinicians and visual scientists.

Our findings from the two experiments suggest that (1) centrally-weighted thalamic patterns allow relatively few phosphenes to produce acuity performance at thresholds for legal blindness, (2) for a given contact count, higher acuity than predicted by our simulations might be available to an implanted patient due to substantial and rapid learning effects, (3) pattern matching strategies are highly effective at low phosphene counts, even though direct perception of letterform shapes requires substantially more phosphenes, and (4) our novel letter recognition paradigm provides acuity assessments that are highly congruent to measurements from the traditional Snellen chart test and thus establishes a solid foundation for inter-species and inter-laboratory comparisons.

## Methods

### 2.1 Subjects

A total of thirty-seven volunteer subjects with self-reported normal or corrected-to-normal vision were recruited from students and staff at the Massachusetts General Hospital or via advertisement to participate in this research. Sixteen of them (10 M, 6 F; 21–72 years old) volunteered for the Primary experiment while twenty-one (9 M, 12 F; 21–56 years old) volunteered for the Validation experiment.

#### 2.1.1 Ethics Statement

The research protocol used for this study was approved by the Partners Human Research Committee, the institutional review board (IRB) that oversees human research at the Massachusetts General Hospital. As this study was classified as a minimal risk experiment by the IRB, approval was given for verbal consent, as opposed to written consent, to prevent collecting any personally-identifying information. Consent was implied by the existence of a data record. All data were analyzed anonymously.

### 2.2 Apparatus

**Figure 1:**
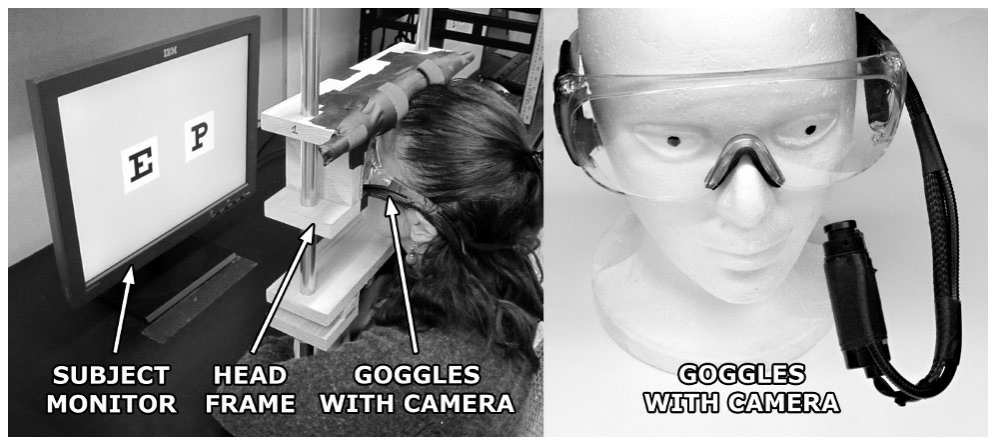
Photographs of the apparatus. A subject is shown performing the experiment (left) along with a close-up of the goggles with eye tracking camera (right). In use, the head frame is adjusted to the seated height of each subject, and the monitor position adjusted to maintain a consistent distance to each subject's eyes. The base of the head frame is mechanically secured to the desk, but the upper part can be raised or lowered. The camera is mounted on a flexible arm that holds position once adjusted for a close-up view of the subject's eye. Experiment workstations and experimentor controls are not shown.

The experimental system (Figure 1) consisted of a wooden frame used for subject head stabilization, a small video camera to measure subject eye position, a set of safety goggles to hold the video camera in fixed relation to the eye, a stimulus computer monitor, and three workstations to run the simulation and capture data. Monocular gaze location was recorded at 30 Hz (PC-60, Arrington Research, Scottsdale, Arizona, 0.5° accuracy, 0.15° resolution).

### 2.3 The Letter Recognition Paradigm

#### 2.3.1 Multiple-Optotype Two-Alternative Forced Choice Task

**Figure 2:**
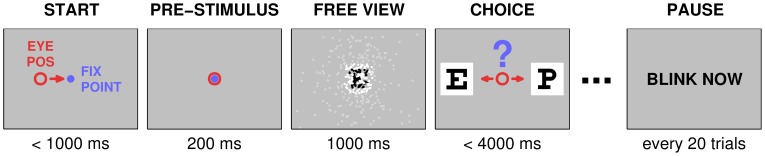
Letter recognition task. Each gray rectangle represents the image shown on the computer monitor during one phase of a trial. Subject eye position is shown as a red circle. The Free View period is represented here under simulated thalamic prosthetic vision as in the Primary experiment: each dot (white, gray, black) is one simulated phosphene; black and white dots represent phosphenes that were presented to the subject while gray ones were not presented and are shown only to indicate how pattern density decreases with eccentricity and that phosphenes spanned the entire visual field (see Section 2.5). During the Validation experiment, cues presented in the Free View period were shown in the clear, rather than through a phosphene field, but the task was otherwise identical.

Subjects performed the *letter recognition task*, a multiple-optotype two-alternative forced choice paradigm based on the Snellen eye chart task. In each trial, a sample letter (*cue*) was presented briefly, and followed by two alternative letters (*targets*), one matching the cue, and one a distractor (Figure 2). Importantly, when starting each trial, the subjects had no prior knowledge of the cue or the pair of alternatives they would eventually be shown. The cue was presented under simulated thalamic prosthetic vision conditions while targets were presented in the clear.

Each trial consisted of a sequence of images presented to the subject in four ordered phases: *Start*, *Pre-Stimulus*, *Free View*, and *Choice*. Trials started with a central fixation dot appearing on the monitor (Start phase). Subjects were required to maintain fixation until dot disappearance (Pre-Stimulus phase), after which the cue was displayed (Free View phase). Next, the cue was extinguished and two targets were presented (Choice phase), indicating subjects were to identify the target matching the cue and communicate their selection by holding fixation on one of the targets. Subjects were instructed to guess when they were uncertain, and not to be concerned by any mistakes they might make. Automated, non-verbal correct/incorrect auditory feedback was given at the end of each completed trial. To help avoid participant fatigue, there was a brief break between blocks, and, within each block, a pause was automatically provided every 20 trials where either an explicit instruction to blink, a humorous aphorism, or a short joke was displayed. If subjects either failed to initially engage at the Start phase of a trial, or failed to make a selection before the end of the Choice phase, the trial was aborted and repeated until successfully completed. The sequence of trials was pre-computed to fully balance and interleave stimulus conditions. Experiment operation, including behavioral control, was entirely automated once each block commenced.

**Figure 3:**
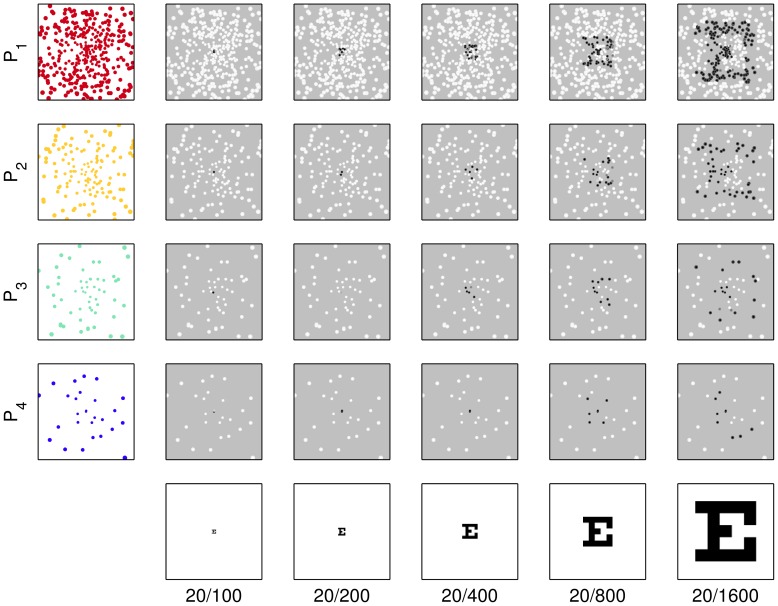
Stimulus combinations. The combinations of optotype size F_1_–F_5_, Snellen 20/100 through 20/1600 (horizontal direction) and phosphene pattern P_1_–P_4_ (vertical direction) are shown in this collection of snapshots of the center part of the subject display during the Free View phase. Parameter values were selected such that it was impossible for subjects to identify letters with the lowest resolution phosphene pattern viewing the smallest optotypes (lower left subfigure) to provide a negative control, and quite easy with the highest resolution pattern viewing the largest optotypes (upper right subfigure) to provide a positive control. Snapshots shown are for gaze positions at the center of the monitor. Only the centralmost 10° of stimuli are shown; the remainder of the screen would be uniformly gray. Stimuli, animated through the gaze-contingent mechanism, were more readily identifiable than the static images above might suggest.

Over the set of trials for each subject, five parameters were varied in balanced fashion: the cue letter during the Free View period, the *distractor* target during the Choice period, whether the *matching* target appeared on the left or right (*match position*) during the Choice period, the font size of the letters, and the phosphene pattern density. The first three parameters were to avoid unintended bias, the last two (*font size* and *pattern resolution*) were the main experimental parameters (Tables 1 and 2). The cue letters were presented at five carefully selected font sizes, *F_1_* through *F_5_*, corresponding to Snellen optotypes for 20/100 through 20/1600 by factors of two, and rendered on 10°×10° white squares (Figure 3). The phosphene patterns were selected based on practicality issues as analyzed and discussed in a previous report [18], starting from a spacing (1000 um) and count (110 electrodes) that is typical of contemporary bundle and array electrodes, and continuing for three steps that approximately double the count each time (Table 2).

**Table 1:** Primary experiment optotype sizes.

*Optotype Size*	*LogMAR*	*Snellen*
F_1_	0.70	20/100
F_2_	1.00	20/200
F_3_	1.30	20/400
F_4_	1.60	20/800
F_5_	1.90	20/1600

Snellen and equivalent LogMAR visual acuity values of the optotype sizes used in the Primary experiment.

**Table 2:** Phosphene patterns.

*Phosphene Pattern*	*Electrode Spacing (*µ*m)*	*Total Count (electrodes)*	*Count in Central 10*° *(electrodes)*
P_1_	400	1700	370
P_2_	600	530	110
P_3_	800	200	45
P_4_	1000	110	25

Numeric details of the experimental parameter of phosphene count. Electrode spacing refers to the distance between electrode tips implanted in LGN tissue in a 3D regular grid pattern that will produce a center-weighted phosphene pattern in visual space [27]. Total electrode count includes electrodes that will generate phosphenes anywhere in the entire visual field, most of which would not be active in the Primary experiment, whereas the count within 10 degrees is for those electrodes generating phosphenes that lie within the central part of visual space corresponding to the approximate location of the letterform stimuli in this report.

#### 2.3.2 Cue, Matching, Distractor Stimuli

Nine standard Snellen optotypes were employed to generate the letter stimuli (cue and targets): C, D, E, F, L, O, P, T, and Z. For each cue letter, three distractors were chosen according to the criteria of legibility, similarity and balance (Table 3); legibility and similarity were based on the investigation by Hetherington [22] on shapes and familiarity of the letters of the Snellen chart, and balance was maintained by ensuring each letter was used as cue and distractor the same number of times. Only three distractors were used for each cue to limit the total number of trials.

**Table 3:** Cues and distractors.

*Cue Letter*	*Distractor Letters*		
C	D	E	O
D	C	O	P
E	C	F	P
F	E	P	T
L	T	Z	O
O	C	D	L
P	D	F	Z
T	F	L	Z
Z	E	L	T

The nine letters used from the Snellen set are shown paired with their three distractors. Each letter appears in the table once as a cue, and three times as a distractor. Each trial uses one of the cues and one of the three distractors associated with that cue. Distractors were selected to match cues in identification difficulty, and to form a closed, balanced set. The letter recognition paradigm involves a total of twenty seven randomly presented alternative choice combinations.

#### 2.3.3 Measurement of Visual Acuity

In the Snellen fractional notation, visual acuity is determined by the ratio of the testing distance to the distance at which the smallest optotypes from the chart that a subject can reliably read would subtend 5 minutes of arc (e.g., the comparatively large 20/400 optotypes would subtend the same visual angle when placed at 400 feet as the reference 20/20 optotypes do at the testing distance of 20 feet). The Snellen score is alternately expressed as the logarithm of the Minimum Angle of Resolution (LogMAR), with a straightforward conversion between the two scales (*m* = −1 • log_10_(*f*) where *f* is the Snellen fraction considered as a numeric value, and *m* is the LogMAR equivalent).

### 2.4 Simulating Thalamic Prosthetic Vision

#### 2.4.1 Phosphene Patterns

Four phosphene patterns, P1 through P4, were used during the Free View period. The center-weighted patterns were generated by simulating four different placements of sets of electrode tips in monkey LGN as detailed in a previous report [18]. The patterns differed in electrode count, ranging from high to low (Table 2). As each individual electrode tip was assumed to generate one independent phosphene at a location in the visual field depending on the electrode's physical location within the retinotopic map of LGN [23], each set of electrodes formed a full-field phosphene pattern highly weighted toward the center of the visual field. In contrast, the equivalent simulation for the retina would produce evenly spaced sets of phosphenes rather than centrally-weighted ones [5].

#### 2.4.2 Generation of Gaze-Contingent Prosthetic Vision Images

The Free View period of the task featured a simulation of prosthetic vision that was central to the experiment. To create the *gaze-contingent* images in the simulation, the set of phosphenes for a given trial was displayed on the subject monitor in such a way as to track the current gaze direction, essentially stabilizing the phosphene locations on the retina. Computationally, phosphenes were treated like holes in a virtual, opaque masking screen placed between the subject and the image to be displayed on the computer monitor. The masking screen was linked to follow the subject's eye positions such that, since the image behind the mask did not move, different parts of the image were revealed through the set of virtual holes with each gaze shift. This was informally reported as a natural-feeling process.

### 2.5 Primary Experiment

#### 2.5.1 Procedure

Before each session, a preliminary fitting and calibration was performed. Each subject was seated in front of the apparatus; the head frame was adjusted to suit, the set of safety glasses equipped with the eye camera fitted, and the subject asked to lean their forehead against the frame and to keep their head still. Display viewing distance was adjusted to be 40 cm, and display height was adjusted so that a relaxed gaze position was at the center of the monitor. The eye-tracking system was adjusted to obtain stable pupil tracking and calibrated as the subject fixated nine sequentially displayed points spanning the stimulus monitor.

The subject then performed the letter recognition task presented in blocks of trials. A total of 1080 trials (4 pattern resolutions×5 font sizes×9 optotypes×3 distractors×2 match positions) was collected in blocks of 180 to 200, presented in a precomputed balanced, interleaved sequence. Each block required typically 15 min to complete and a short pause was given between blocks to limit errors caused by fatigue.

During the Free View period of the task, the image presented on the subject monitor was updated on a frame-by-frame basis. Prior to each trial, the appropriate pre-computed static *letter image* of a black letter on white background and one of the four sets of phosphenes were loaded. Then, for each video refresh of the subject monitor (Model L200p, IBM, Armonk, NY; 800 by 600 pixels at 75 Hz frame rate), a new *subject frame* was constructed, starting with a uniform 70% gray background. The set of phosphene positions was translated to center on the subject's instantaneous gaze position and then used as a mask on the letter image that was fixed in virtual space at the middle of the monitor. Phosphenes with positions that overlaid the letter image were rendered on the subject frame as either black or white 2-D Gaussians depending on the intensity at the corresponding pixel of the letter image. The size of each displayed phosphene was determined by apparatus geometry, retinotopic eccentricity [24], and reports from the literature [25], [26], [27], [28]; phosphenes were never smaller than one pixel, and became larger with increasing eccentricity, being about 0.5 degrees in visual angle at 10 degrees from the fovea. Once all phosphenes had been processed, the assembled image was presented on the subject monitor, synchronized to the video frame update. Since the cue covered only a visual angle of 10°×10° at the center of the monitor, only about one quarter of phosphenes in the pattern under test were typically engaged, although the exact number varied from trial to trial due to the influence of instantaneous subject gaze location on presented stimuli (Figure 3). The subject was allowed to free view the simulated image for a fixed period of 1,000 ms.

#### 2.5.2 Data Analysis

Two main experimental factors were considered, font size and pattern resolution (defined above as the size of the letters, and the number of simulated contacts of the thalamic visual prosthesis, respectively). Each subject's per-trial responses were pooled by font size F*i* and phosphene pattern P*j*, and analyzed for percentage of correct responses, or *hit rate*, and latency to selection initiation, or *response time*. Only completed trials were included in the analysis, the first 40 of which were discarded and excluded from all subsequent analysis to eliminate effects from startup transients.

A suite of computations was performed on the data collected from each subject. For each font size/pattern resolution combination F*_i_*:P*_j_*, or *condition*, visual acuity was assessed by the percentage of completed trials that were answered correctly, or *subject hit rate*. Next, for each condition, the average of the response times of correct trials, *subject response time*, was calculated after discarding outliers that were below 150 ms, or above 1.5 times the inter-quartile range beyond the third quartile. Finally, changes in performance were analyzed by comparing hit rates and means of response times of the last segment of 200 trials (10 per condition) to those of the first segment of 200 trials immediately following the initial 40 discarded trials.

As the distribution of response times skewed toward zero, careful consideration was given to outlier elimination in the calculations above. Two methods were compared, logarithmic transformation and median-referenced cutoff with thresholds as given above (see [29] for a review of different approaches). The median method typically rejected one additional datum per subject than the logarithmic method. For simplicity, the median method was used, excluding 0.35% of the cases from further analyses. Excluded data points were evenly distributed over experimental conditions and individuals.

Visual acuity for the population of subjects was then measured in two ways for each phosphene pattern. First, the smallest font size was identified for which population performance was statistically above chance, or *first significant deviation* (FSD) with one-sample T-tests for each font size F*_i_*. Second, a psychometric curve was fitted to the population mean hit rate and used to determine an interpolated font size for 75% performance, or *interpolated 75%* (i75), at the mid-point of the psychometric fit.

Two-way repeated measure ANOVA was performed to test the significance between pattern resolution and font size for subject hit rate and subject response time; contrast tests and paired T-tests were used within factor conditions and to evaluate performance changes. The ANOVA was corrected using Greenhouse-Geisser estimates when Mauchly's sphericity was violated. *Population hit rate* and *population response time* were calculated by averaging per-subject hit rate and response time, respectively, over all 16 subjects. As there were typically 50 trials per condition, a significance level of *p* = 0.02 was imposed to avoid false positives. SPSS Statistic 19 (2012, IBM, USA) was used for advanced statistical calculations.

### 2.6 Validation Experiment

To validate the letter recognition paradigm, a secondary experiment was performed to directly compare the Snellen test with its multiple-optotype two-alternative forced choice equivalent. Subjects first performed a standard chart-based Snellen visual acuity test and then carried out the same letter recognition paradigm presented above, except that cue optotypes (as well as targets) were shown in the clear, and font sizes were adjusted to replicate the Snellen chart.

#### 2.6.1 Motivations

Although our letter recognition paradigm uses the same optotypes as the Snellen acuity test, we needed to verify that the differences in test design would not result in differences in assessments. The Validation experiment was therefore designed to explore potential dissimilarities between the two methods and, importantly, establish the veracity of results from the new paradigm as we look to support its use across species and laboratories.

#### 2.6.2 Procedure, Validation Experiment

**Figure 4:**
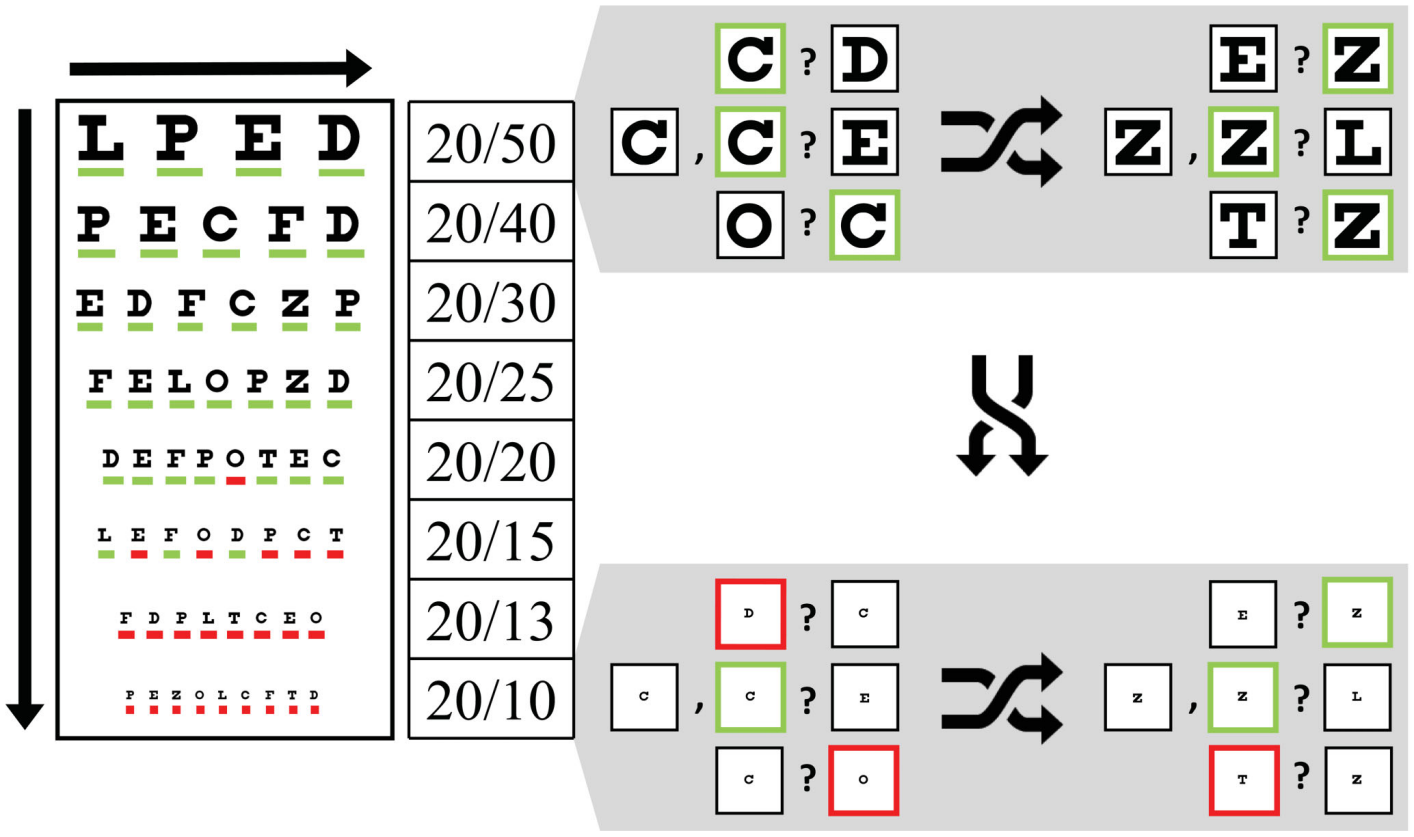
Comparison between paradigms. Testing using the Snellen chart (left) proceeds top to bottom, and left to right, with the subject calling out each letter on successive rows, or declaring their inability to do so. Testing started with the 20/50 line as shown here, which is the fourth line of standard charts. Testing using our letter recognition task (right) is performed in a balanced, randomly interleaved pattern; the figure, designed to show the equivalence between tests at given acuities, uses shuffle arrows to imply the interleaving. Trial conditions are represented as a cue letter followed by the two alternatives. Red and Green highlights illustrate single letter scoring (correct/incorrect) during an example data collection session where the subject was assessed with 20/18 on the Snellen task and 20/20 on the letter recognition task.

Twenty one subjects (Section 2.1) performed two acuity tests (Figure 4), a traditional Snellen chart test and then a variant of the letter recognition task. First, subjects stood 20 feet from a wall-mounted Snellen eye chart and were administered the acuity test starting on the fourth line of the chart (20/50, a level well above the predicted acuity threshold but below the top lines to reduce the number of trials necessary in the second part of this experiment). Each subject was instructed to call out the identity of each successive letter, one row at a time. For each row, the number of letters correctly identified was recorded. Testing proceeded at a pace set by the subject.

Next, subjects performed the letter recognition task (Section 2.3). Subjects were again fitted and the eye tracker calibrated as described above, but in this experiment the monitor was placed at a distance of 105 cm, and optotypes were used that replicated the eight Snellen chart acuity levels 20/10 through 20/50 (Table 4). In each trial, the font size, cue, distractor, and match position were presented in a balanced, interleaved sequence as before, however, in the Free View phase, the cue was presented in clear, unmasked form as there were no phosphene patterns under test. The main experimental parameter was font size, with a total of about 400 trials collected per subject (8 font sizes×9 optotypes×3 distractors×2 match positions). Data collection for each subject typically lasted half an hour with one or two breaks.

**Table 4:** Validation experiment optotype sizes.

*Optotype (Font) Size*	*LogMAR*	*Snellen*
VF_1_	−0.30	20/10
VF_2_	−0.19	20/13
VF_3_	−0.13	20/15
VF_4_	0.00	20/20
VF_5_	0.10	20/25
VF_6_	0.18	20/30
VF_7_	0.30	20/40
VF_8_	0.40	20/50

Corresponding Snellen and LogMAR visual acuity values of optotypes used in the Validation experiment. These irregularly spaced sizes correspond to the lower eight lines of the standard Snellen chart.

#### 2.6.3 Data Analysis, Validation Experiment

Subject performance was analyzed in two ways: first, the hit rates for each font size were compared across the population; and second, the differences in visual acuity scores from the two methods were examined for each subject.

In the first analysis, the hit rate results from the two tests were fitted with psychometric curves. Data from the letter recognition task were normalized for each subject to a [0–1] range, while for the Snellen task, a percent-correct-per-line scoring method was used that resulted in an equivalent [0–100%] range. A repeated measure generalized linear model was used to assess differences between the two data sets. Font size and type of acuity test were used as independent variables while hit rate was used as the dependent variable.

In the second analysis, agreement between visual acuity scores given by the two tasks was evaluated using Bland–Altman analysis [30]. For the Snellen chart, the score was determined by the smallest line for which more than half of the optotypes were correctly identified plus the fraction of additional optotypes correctly identified on the next smaller row. For the letter recognition task, i75 acuity was used.

The International Council of Ophthalmology suggests that it is clinically most informative to record the exact responses when the subjects achieve partial success at different size levels of a chart [31]. Therefore, visual acuity scores in the Snellen chart were determined using the rigorous single letter scoring method described above. Moreover, since the Snellen chart test was performed only once in our experiment, it was crucial to employ a scoring rule that minimizes Test-Retest variability. As reported by Raasch, *et al.* [32], letter-by-letter scoring rules yield significantly lower standard error than whole-line scoring rules.

## Results

### 3.1 Primary Experiment

#### 3.1.1 Hit Rate

**Figure 5:**
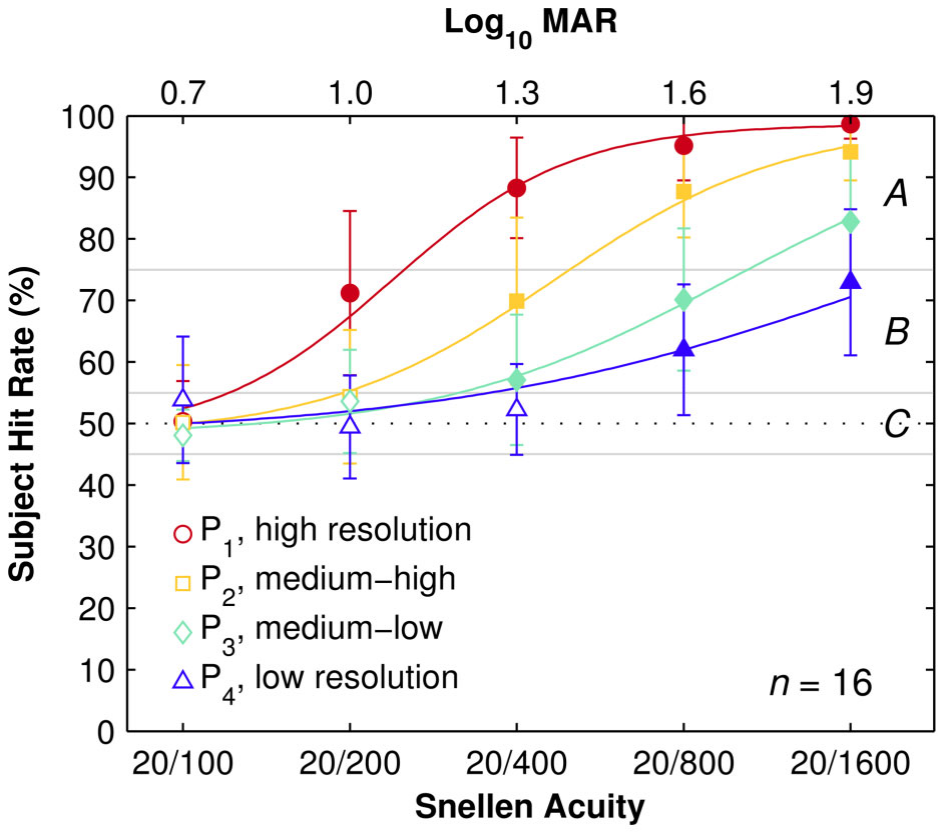
Hit rate results. Hit rate as function of font size, grouped by pattern resolution. For each condition, symbols indicate population mean and error bars, standard deviation. Each trace represents the fitted curves of subject hit rate for each pattern resolution (mean deviation below 1.2%). Font size is shown on the horizontal axis. Filled symbols are significantly above chance. The letters *A*, *B*, and *C* indicate the High-Performance, Mid-Range, and Chance/Low-Performance ranges, respectively (see main text). Subjects were not significantly above chance for all phosphene patterns for the smallest optotypes (F_1_, Snellen 20/100), with population hit rate increasing with both pattern resolution and optotype size.

For all phosphene patterns Pi, subjects performed not different from chance (50%) with Snellen 20/100 optotypes (F1, smallest font size) while performance rose to nearly perfect (100%) with Snellen 20/1600 optotypes (F4, largest font size). The two acuity measurements, FSD and i75, for each pattern Pi are listed in Table 5. As expected by inspection of Figure 5, ANOVA analysis confirms a significant main effect of font size (F(1.8, 25) = 192, p<0.001) and pattern resolution (F(2.3, 32) = 169, p<0.001) on subject hit rate. The significant interaction between font size and pattern resolution (F(12, 168) = 16, p<0.001) on subject hit rate indicates that they are not independent (see Discussion). Counting the number of phosphenes from each pattern within a circle at the origin with diameter five times the angle implied by the LogMAR levels, equivalent to the five dark/light phases in a Snellen E, we find there are 20±2 phosphenes required to reliably recognize a given optotype size at i75 levels (see Discussion).

**Table 5:** Visual acuity values for each phosphene pattern.

*Phosphene Pattern*	*Central Phosphenes (count)*	*FSD Acuity (LogMAR, Snellen)*		*i75 Acuity (LogMAR±95% confidence, Snellen)*	
P_1_	370	1.00	20/200	1.08±0.09	20/240
P_2_	110	1.30	20/400	1.37±0.08	20/470
P_3_	45	1.30	20/400	1.68±0.09	20/960
P_4_	25	1.60	20/800	2.00±0.13	20/2000

For each phosphene pattern under test, P*_i_*, the number of phosphenes in the central part of vision is shown, followed by the population mean acuity using the two criteria of first significant deviation from chance, and interpolated 75% hit rate (see main text). The FSD acuity corresponds to the stimuli where the population response first differs significantly from chance (*p*<0.001 for all except P_3_ for which *p*<0.02), whereas the i75 acuity corresponds to the 75% performance level of a sigmoid fitted to the population data. Uncertainties shown for i75 values represent 95% confidence ranges.

#### 3.1.2 Response Time

**Figure 6:**
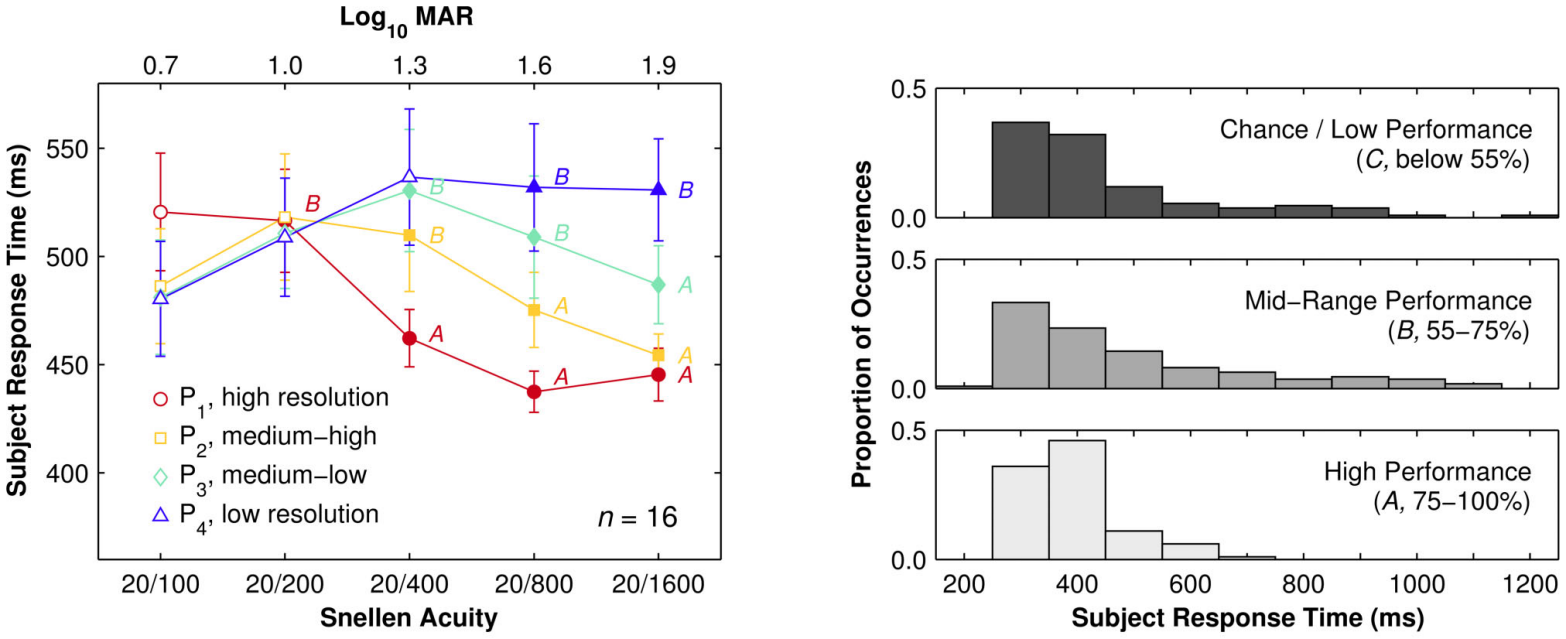
Response time results. (LEFT) Response time as a function of font size, grouped by pattern resolution. Population mean (symbols) and standard deviation (error bars) of subject average response time for correct trials for each condition. Filled symbols indicate conditions with statistically significant population hit rates. Annotations of *A* and *B* symbols indicate conditions with hit rates in Mid-Range and High-Performance ranges, respectively, while unannotated symbols are conditions in the Chance/Low-Performance range. High-Performance conditions all have faster population response times than Mid-Range conditions, whereas Chance conditions have intermediate values that are affected by font size but not pattern resolution, together suggesting that two different perceptual mechanisms were involved. (RIGHT) Distribution of response time broken down by hit-rate performance ranges. The three graphs depict the relative occurrences for mean response times per condition binned to 100 ms for data from Chance/Low-Performance (upper), Mid-Range (middle), and High-Performance (lower) conditions. A Kolmogorov-Smirnov test found no significant difference between the Low-Performance and Mid-Range data (*p* = 0.47), while a significant difference was found between the Mid-Range and High-Performance data (*p*<0.01). There is a strong functional difference between performing at versus above chance, guessing versus knowing the answer, despite no significant difference found between the respective distributions. In contrast, there is only a weak functional difference between the upper two performance ranges, reflecting a sliding degree of task difficulty, but the significant difference in distributions suggests the emergence of a distinct mechanism at upper performance levels.

Task difficulty and the underlying cognitive processes used during a perceptual decision-making task can be deduced from subject response time [33]. Figure 6 illustrates the population response time as a function of font size over all subjects. There was not a significant main effect of font size on subject response time (F(4, 60) = 1.3, p = 0.29), but there was a significant main effect of pattern resolution (F(3, 45) = 3.5, p<0.05). Moreover there was a significant interaction between font size and pattern resolution (F(12.0, 180) = 4.6, p<0.001).

We further analyzed the population response time results by grouping values by hit rate, according to the three ranges of *Chance/Low-Performance* (50–55%), ***Mid-Range*** **(55–**75%) and *High-Performance* (75–100%). The population response time significantly decreased for High-Performance conditions (*t*(89) = 3.5, *p*<0.001) but increased for Mid-Range conditions (*t*(89) = 2.6, *p*<0.01) compared to the Chance range. Specifically, High-Performance conditions are significantly different from Mid-Range conditions (*F*(1, 15) = 5.3, *p*<0.05). For F2 optotypes (Snellen 20/200; LogMAR 1.00) the population response times are not significantly different (*p*>0.36). Based on this observation, we additionally computed the Pearson's correlation between per-subject hit rate and response time, which indicated a significant linear correlation between the two (*r* ^2^ = −0.69, p<0.001). As will be argued in the Discussion (Section 4.3), response time differences might indicate multiple mechanisms were used in selection of the matching target in conditions with hit rates above chance (see also Figure 6, right).

#### 3.1.3 Within Subject Performance

**Figure 7:**
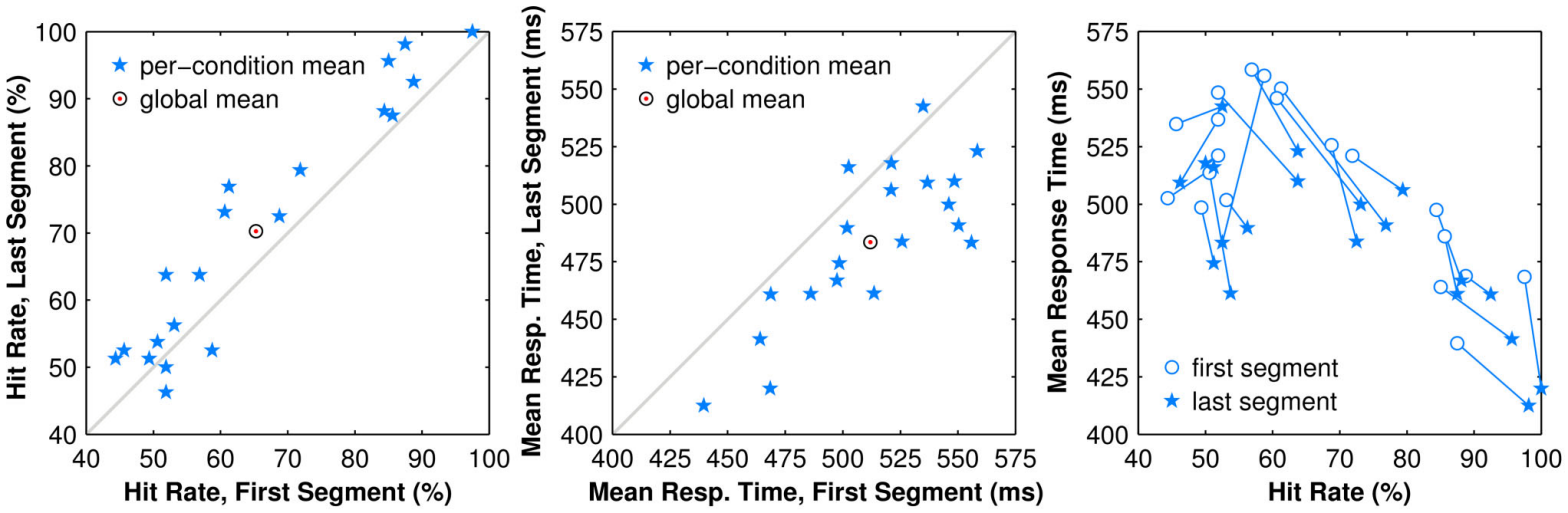
Learning effects. Scatter plots of population hit rate and response time in the first versus last segment of trials. (LEFT) Population hit rates split by condition (stars) and overall mean (bulls eye). Data are above the line of equality as subjects perform more accurately in the last 200 trials (10 per condition) than in the first 200 trials. (CENTER) Population response time for each condition (stars) and overall mean (bulls eye). Nearly every datum is below the line of equality as subjects have faster responses in the last 200 trials than in the first 200. (RIGHT) Combining population response times and hit rates from the two previous graphs with linkages between first (open circles) and last (filled stars) segments reveals two distinct spans of behavior, one at low hit rates that is more disorganized, and one at higher hit rates that displays strong structure. The lower hit rates correspond to the Chance regime where subjects are more likely to be guessing; the higher hit rates correspond to the Mid-Range and High-Performance regimes. The largest improvements, as shown by the longest linkages, cluster around the threshold between Chance and Mid-Range regimes.

The performance of each subject was also analyzed for changes in hit rate and per-trial response time within the experimental duration. For each subject performance in the first and last 200 trials (approximately the first and last 1/5th; the first 40 trials remained discarded) were compared. As conditions were presented in a fully balanced sequence, segments of 200 trials represent an equal number of trials in each condition. For hit rate, subjects were significantly more accurate at the end versus the start of the experiment overall (71% vs. 65%; *t*(15) = −2.5, *p*<0.03), with the effect being largest for Mid-Range conditions (72% vs. 63%; *t*(5) = −5.1, *p*<0.04) as compared to High-Performance (92% vs. 86%; *t*(5) = −3.4, *p*<0.01) and Chance (52% vs. 50%; *t*(7) = −1.2; *p* = 0.26) conditions (Figure 7). For response time, subjects were significantly faster in the last versus first segment (483±34 vs. 511±34 ms; *t*(19) = 5.72; *p*<0.001; see Figure 7); however there were no significant differences between conditions within each segment in either case (*F*(1.9, 28) = 2.1, *p* = 0.14). The overall response time improvement without a condition-specific differential improvement suggests a global learning effect produced by training.

### 3.2 Validation Experiment

**Figure 8:**
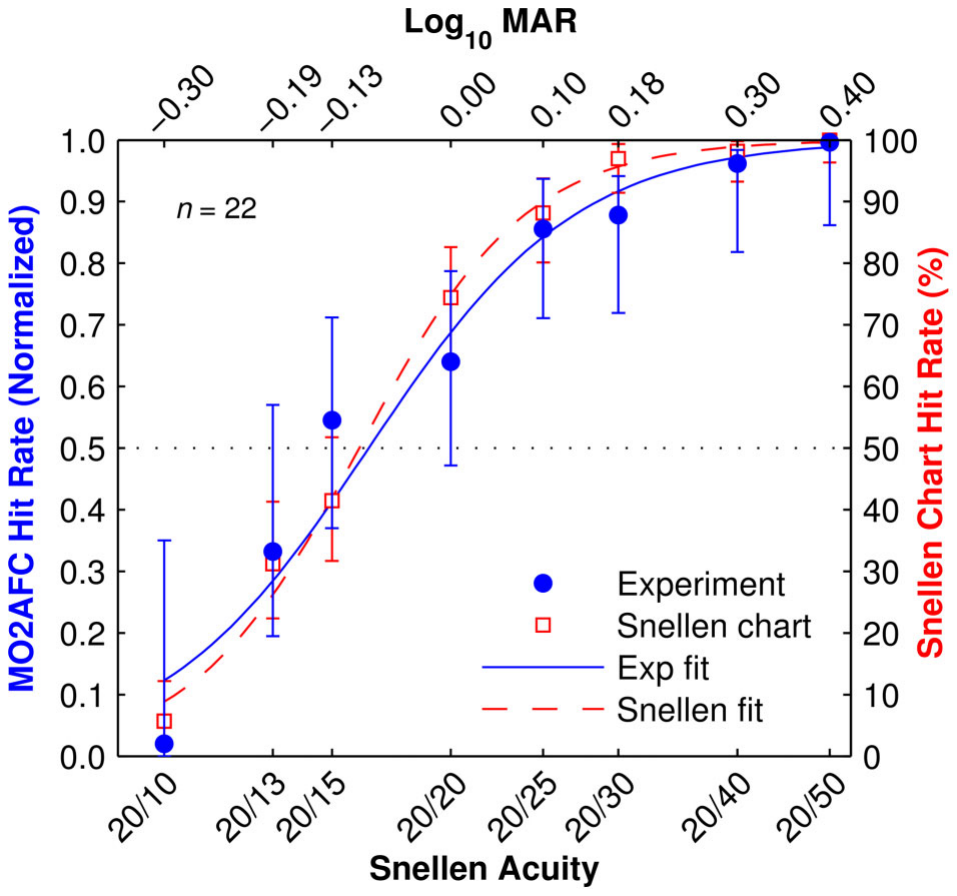
Validation results. Hit rate as function of font sizes (translated to the equivalent acuity) for the standard Snellen chart test (red unfilled squares) and the multiple-optotype two alternative forced choice (MO2AFC) letter recognition task (blue filled circles). Error bars are two-sigma confidence levels. Smooth traces are sigmoid fits to each data set. Uneven spacing across the horizontal axis reflects the step sizes between lines in the standard Snellen chart. Results from the letter recognition task have been normalized to span [0–1], an equivalent range as the Snellen chart test (0%–100%), for ease of comparison. The two curves are in high agreement, although there appears to be a trend to a shallower transition with the letter recognition task, possibly due to uncorrected false negatives at the upper end of the range.

**Figure 9:**
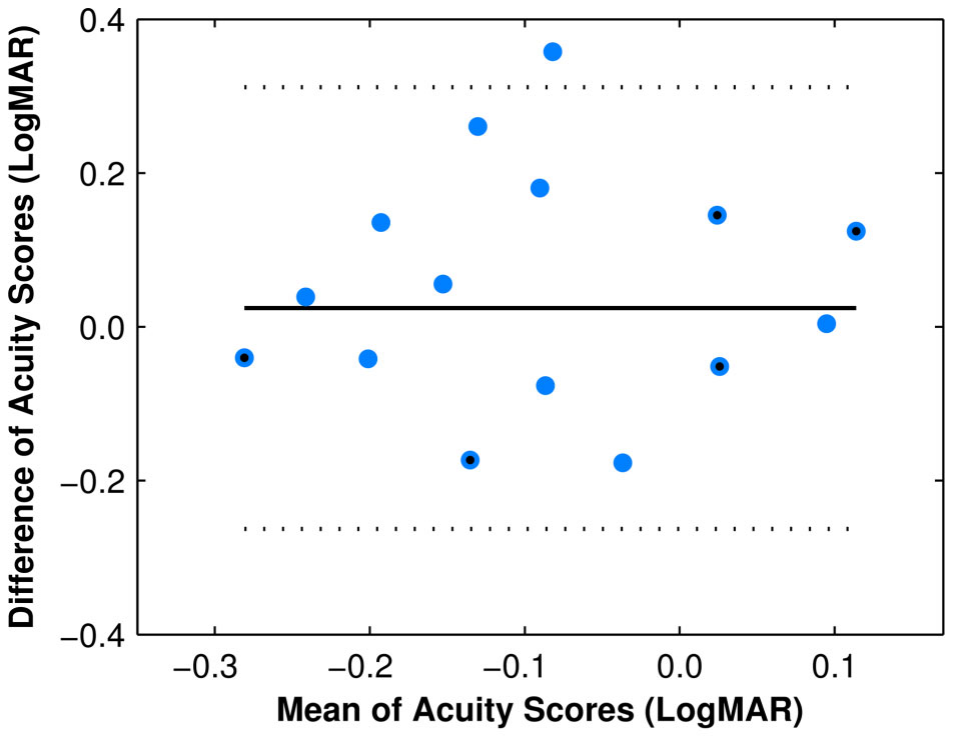
Validation analysis. Bland-Altman plot of the differences in visual acuity scores between our letter recognition task and the Snellen chart test. The vertical axis displays the difference between measurements on both tasks, and the horizontal axis displays the mean acuity score value between the tasks. The solid line is the mean difference in LogMAR acuity (−0.02), and the area within the dotted lines represents the limits of agreement between LogMAR scores in the two visual acuity tasks. Each point represents the results from a single subject (blue circles), although some are overlapped (blue circles with black centers).

The hit rate results of the population of subjects for each font size of the letter recognition task and the Snellen chart revealed no significant differences between the two by repeated-measure generalized linear model (*p*>0.5). The population visual acuity at a 50% threshold *T* was estimated at the mid-point of the psychometric curves (equivalent to the i75 measurement described above) as *T*_Snellen_ = −0.08 LogMAR, *T*_Validation_ = −0.09 LogMAR, for a difference of 0.01 LogMAR (Figure 8). Bland-Altman analysis, evaluating differences between visual acuity scores between the two acuity tests plotted against their mean, shows a mean difference of 0.02 LogMAR (Figure 9).

## Discussion

### 4.1 Decision Making in Multiple-Alternative Visual Acuity Tasks

Visual acuity is the main measure of visual function in both clinical and research settings. Many kinds of stimuli have been used to measure acuity including gratings, dots, bars and optotypes, and a wide variety of test charts are available. It has been said that, “[t]here are almost as many different visual acuity test charts as there are ophthalmological departments!” [34].

The accuracy in multiple-alternative visual tasks critically depends on several parameters, including mainly: (1) the number of alternatives, (2) the time of exposure to information, (3) the speed-accuracy constraints imposed by the experimenters, and (4) the subject's prior knowledge of stimuli likelihood, if any.

Our paradigm involves multiple optotypes in a two-alternative forced choice task presented in random order. Since we use the entire set of Snellen optotypes, the prior knowledge of possible alternatives before a trial is the same as in the Snellen acuity test. However, during the exposure to the stimulus, while the integration of evidence favoring each alternative remains based on the entire set of optotypes in our paradigm, the Snellen acuity test allows an online visual update of evidence when larger optotypes are available on the chart for comparison. Without any time constraint, the subject's decision is then made when sufficient evidence has accumulated favoring one alternative over the others. In our paradigm, however, the cue is presented for a limited period of time and the two possible alternatives are subsequently revealed, also during a limited period of time, when the stimulus is no longer available for comparison. The subject's decision is made when sufficient evidence has accumulated from these two consecutive time-constrained phases.

A well-known result in psychophysics is that the time taken to judge between alternatives is proportional to the amount of stimulus information to be processed according to Hick's law [35]. While traditional investigators of Hick's Law have emphasized error-free responding by forcing observers to process all of the information provided by the stimulus, it has often been found that when speed-accuracy tradeoffs are imposed, the response accuracy declines steadily as the number of choice alternatives increases.

Despite methodological differences between our letter recognition task and the standard Snellen acuity test, we found no significant difference in the letter-by-letter scoring results. We argue that the similarity of outcomes can be explained by two main factors. First, the initial decision of the subject results from the information processed during the exposure to the cue, for which prior knowledge is identical in both paradigms as they share the same set of optotypes. Second, a brief time of exposure (1,000 ms) followed by two alternatives in our paradigm could have compensated for an unlimited time of exposure with more than two alternatives in the Snellen chart test, some of which are visually available for additional integration of information.

While the close correspondence of results from the two approaches provides a solid foundation for use of the task in comparisons between laboratories, it also carries implications for the threshold criterion used to measure visual acuity in our letter recognition task. Specifically, the results indicate that the 75% hit rate criterion is more clinically relevant than the first significant deviation above chance. Baseline performance on two-alternative forced choice tasks like our letter recognition task is 50%, whereas baseline performance on the Snellen chart task is 0%, and standard threshold is 50%. Projecting the Snellen threshold to the letter recognition task yields the 75% threshold (i75), the level at which good agreement was found in the Validation experiment. Importantly, that projection does not match FSD criteria often reported in the literature, suggesting that more accurate figures will result from using 75% thresholds in future reports.

### 4.2 Comparison to Other Simulations of Visual Acuity Studies

Due to differences in task design, phosphene simulation and phosphene patterns used in other studies, a direct comparison between the results from the Primary experiment and related work is difficult. As a preliminary example, the majority of published studies employed uniformly grey colored pixels or dots to simulate phosphene appearance [8], [7], [12] with only one study using Gaussian profiles [9], similar to the present work. More importantly, and as discussed above, when comparing different paradigms the complex interaction of several experimental design factors influencing the error-rate of multiple-alternative forced choice visual tasks must be considered. In the visual prosthesis literature, visual acuity was mainly tested using the Tumbling E or the Landolt C paradigms. In these studies, the subjects were informed of the possible alternatives they would be shown (four orientations for the Tumbling E and nine for the Landolt C) before starting the experiment. Moreover, as noted by Chen *et al.* [9], the Tumbling E's square appearance was distorted to a rectangle in earlier studies by Cha *et al.* [8] and Hayes *et al.* [12], making obvious the differentiation between up-down oriented Es and left-right oriented Es and thus reducing the exercise from a 4AFC task to a 2AFC task. In the present nine letter 2AFC task there are 27 possible cue-distractor pairs and the subject is naïve to the pair selection, making the task more difficult to perform than the others cited above.

Therefore, only an indirect comparison can be made between studies, achieved by extrapolating data from the literature to estimate equivalent performance under conditions used in the present study. Cha and colleagues [8], using a Tumbling E task, reported 20/100 acuity at FSD for a regular matrix of 100 phosphenes spanning 1.7° by 1.7° of visual angle; extrapolating the regular array out to 10° by 10° yields about 3460 electrodes. Hayes and colleagues [12] also used Tumbling Es and obtained 20/420 at i75 for 256 evenly spaced electrodes spanning 11.3° by 19.3°, implying about 130 spanning 10° by 10°. Chen and colleagues [9] used a Landolt C task in a simulation of 100 electrodes spanning about 16° by 16°, or about 40 electrodes at 10° by 10°, reporting at i75 level a mean visual acuity of 1.68 LogMAR (Snellen 20/960) for the hexagonal and 1.74 LogMAR (Snellen 20/1100) for the rectangular pattern in the first sessions of their experiment, with an equivalent number of trials as the present report. Similarly, Cai and colleagues [7] showed a mean acuity ranging from 1.36 to 1.55 LogMAR (Snellen 20/460 to 20/710) i75 for 100 electrodes covering 10.8° by 10.8° in a Tumbling E task.

We have extended the difficulty and accuracy of acuity tests by using a brief, fixed identification time, in addition to using a set of nine standard optotypes. In our letter recognition task, optotypes are viewed for a short, fixed time (1,000 ms), whereas other experiments allowed longer periods from 2,000 to 15,000 ms [8], [7] or, more typically, an indefinite viewing period [9], [11], [12], [36], [37], [38], [39], [40]. While the viewing time employed in this study is considered sufficient for reading with natural sight (second-grade children require 720 ms to read a letter while adults require 320 ms [41]), it could be insufficient with artificial sight [38]. Thus, our results most likely reflect minimal visual acuities achievable through thalamic visual prostheses, and should be viewed as conservative estimates in comparison to other reports from the literature.

### 4.3 Response Time and Recall Mechanisms of Prosthetic Vision

To understand response time observations in the Primary experiment, it is important to first understand the two aspects which combine to determine how difficult a given stimulus is to recognize: the visual complexity of the imaged object, and the density of the sampling pattern used. For a given phosphene pattern, visually simpler objects are easier to recognize than more complex ones. Similarly, for a given object, denser phosphene patterns make recognition easier than more sparse ones. Thus, image difficulty varies directly with object simplicity and inversely with pattern resolution, as would be expected by applying Shannon sampling theory to a two-dimensional image with a potentially non-uniform sampling pattern.

The few studies that have specifically investigated response time in a visual prosthesis simulation support the conclusion that response time varies with stimulus difficulty. Thomson and co-workers [42] reported a significant inverse relationship between the number of phosphenes and response time on face recognition. In addition, Guo and colleagues [43] found a small, although not significant, decrease of response time as the number of phosphenes increases in an object recognition task. Yang and colleagues [40] showed response time increased with complexity of Chinese characters and decreased with number of phosphene count. Finally, Dagnelie and colleagues [11] showed that reading speed of single words increased as the number of phosphenes increased or as the font size increased. Taken together, these studies suggest that response time increases with the complexity of the scene or the reduction of phosphene count, the two factors described above that combine to form image difficulty. Our findings support this conclusion while adding details that suggest there are two separate mechanisms in play.

In the Primary experiment, population response times were lowest for High-Performance conditions (those with hit rates above 75%), increased for Chance conditions (50–55%), and were longest for Mid-Range conditions (55–75%). That is, the High-Performance conditions appear to be easier than Chance conditions, and Mid-Range harder than Chance. This counter-intuitive observation can be thought of as subjects answering quickly when identification is easy, giving up after attempting identification and realizing it is impossible, and working hardest under middling conditions. The differences might also be understood as follows. Nobel and Shiffrin [44] demonstrated that cued recall for familiar words — the ability to recollect an object prompted by a verbal or nonverbal cue — requires more time than outright recognition of a word, subsequently modeling cued recall as a serial process and recognition as a parallel one. Despite differences from the Nobel and Shiffrin experiment, we speculate a similar process might occur during the Choice phase in the present study. If the cue letter is identified during the Free View phase we propose that subsequent matching might be made by direct comparison of the internal representation of the letter with the target images presented. If the cue letter has not been identified but the subject was able to acquire clues as to the letter identity, subsequent matching might be made by serial search where each remembered feature is compared to both target letters and the target with highest likelihood chosen. These assumptions then may help explain why Chance conditions are slower than High-Performance conditions yet, surprisingly, faster than Mid-Range conditions: if too few clues were acquired then the subject has fewer comparisons with respect to Mid-Range conditions but more than High-Performance conditions.

Implications of this interpretation are three-fold: (1) post-implant rehabilitation should promote feature extraction more than direct object perception at low relative resolutions; (2) pre-processing filters that extract and emphasize salient clues about object identity may have advantages over more veridical filtering; (3) additional inquiry will be required to determine if this behavior is unique to the thalamic visual prosthesis or is a common phenomenon of all approaches.

### 4.4 Practice Improves Response Time and Hit Rate

Subjects had a significant improvement in response time and hit rate between the beginning and the end of the Primary experiment. This observation is consistent with previous work, such as reported by Thompson, *et al.* [42] and Chen and colleagues [9]. Since response time improvement was a global effect, we might expect that subject ability will increase with longer practice at even the lowest resolutions, as suggested in the previous paragraph. This hypothesis is bolstered by the observation that improvement in hit rate was largest for Mid-Range conditions where even a modest improvement will increase the measured acuity, consistent with previous reports [36], and that there was an improvement for Chance conditions, although it was not significant (Figure 7, right panel). Therefore, visual acuity measurements reported here are likely to improve given sufficient time and practice. Also, as learning was rapid and substantial, we speculate that patients with implanted devices might respond advantageously to new visual modalities that would, for example, present an encoding of the visual scene rather than presenting it pixel-for-pixel. Such methods might take advantage of the brain plasticity to improve learning performance and increase ultimate usability.

### 4.5 Limitations of the Simulation

Limitations of the artificial vision simulation used in the Primary experiment have likely impacted the reported results. For example, the fidelity of the subject image during the Free View period is limited by the resolution of the monitor, affecting small image features more than large ones. The effect would have been more pronounced for the smallest phosphenes, for pairs of phosphenes with small separations, and for the smallest features of each letter, reducing legibility in each case. Results for the F_1_ (Snellen 20/100, LogMAR 0.70) optotypes may have been particularly affected, driving observed performance artificially toward chance. Additionally, uncertainty in eye position due to small head movements, system noise inherent to the eye-tracking method, and overall system latency (estimated as 25–50 ms between gaze shift and image update during cue presentation), introduced spatial noise in the images presented, which has been found to negatively affect subject performance [45], [46]. Finally, eye position was only measured monocularly and a single simulation image was viewed by both eyes; no attempt was made to present independent images to each eye nor to compensate for ocular torsion or vergence. Due to a combination of these factors and others mentioned previously, we speculate that the visual acuity reported here might be an underestimate of the acuity achievable by future thalamic prosthesis recipients.

Beyond these more practical limitations, there is also the question of phosphene independence that has been assumed in the present work (see also 4.6 below). The only published example of patterned stimulation in primate LGN thus far [15] showed evidence for independent control of two phosphenes that were well-resolved by behavioral measures. Nevertheless, confirmation and expansion of that result to a larger set of electrodes is required.

### 4.6 Simulations and Predictions

The reason to establish a prosthesis simulation is to make predictions about potential device designs without incurring the risks and costs associated with a full implementation, some of which may not be justifiable even in an animal model without the confidence of efficacy. The predictions made in this work are based on a simulation of thalamic prosthetic vision that embodies assumptions based on available empirical evidence from experimental thalamic microstimulation [15] and knowledge of the representation of the visual field in LGN [14], [18], [23], including evidence for retinotopic phosphenes [15] that we have presumed requires gaze-contingency to accurately simulate (e.g. [10], [47], [48]). One of the largest uncertainties is whether patterned, concurrent stimulation among multiple thalamic microelectrodes will obey superposition and create a set of phosphenes equivalent to the aggregation of each phosphene generated from stimulating electrodes individually. Contemporary reports from retinal work support this assumption [4], [2], but detailed empirical evidence for LGN microstimulation, once available, will allow for more accurate refinement of the model embodied in our simulation and strengthen its predictions.

To elucidate any underlying mechanism that ties phosphene pattern density to observed acuity, we measured the number of phosphenes in each pattern that would lie within a circle circumscribing each optotype size at i75 performance as described above. Snellen optotypes are designed with feature sizes that are 1/5 the total optotype extent, as typified by the three arms and two spaces in a vertical cut through an E. Given that the phosphene pattern in a visual prosthesis is essentially sampling a 2D image, by Shannon's sampling theory, we would expect 25 points in a uniform pattern to be required to represent an optotype, or, put conversely, the smallest optotype representable would be one that spanned a 5-by-5 regular grid, due to the Nyquist limit. With thalamic phosphene patterns, there are three primary differences: the first is that the sampling is non-uniform, and thus Shannon's theory does not directly apply; the second is that as the task is gaze-contingent, and as subjects have enough time to make two or three fixations during stimulus presentation, they can integrate information from what amounts to multiple samplings; the third is that we do not know if subjects are acting optimally. Nevertheless, when we assessed the number of phosphenes that are likely to carry information useful to the task by counting the number that fall within the area circumscribing optotype size at i75 acuity, we find there is a surprisingly uniform number of phosphenes involved, namely, 20±2 reported above, reflecting the generally better-than-theoretical performance seen in the simulation literature such as reviewed by Chen and colleagues [49]. Thus, we expect that given a phosphene pattern, we can predict the acuity it will afford by measuring the diameter of a circle encompassing the centralmost 20 points.

### 4.7 Cross-Species Applications

When designing the letter recognition task, we strove to make the design compatible with future comparisons with animal models. Visual object recognition is a complex task involving not only the optical capacity for resolving an image and the cognitive ability to identify it, but also the motor ability to respond. A major feature of our design keeps the motor requirement compatible with automated processing and cross-species testing by employing eye position as a behavioral response. Keeping in mind the rich literature in non-human primate behavior using match-to-sample tasks with distorted samples (e.g., [50], [51], [52]), we focused on the multiple optotype two alternative forced choice task as a building block of our paradigm. Our method was shown to be comparable to a standard acuity test in humans, enabling its use in cross-laboratory and clinical comparisons. Although this report contains results from only human subjects, given the additional factors stated above, we speculate that our task will prove useful in cross-species comparisons as well.

## Conclusions

The effect of number of phosphenes on visual acuity, learning rate, and response time during a thalamic prosthetic vision simulation was investigated using a novel letter recognition paradigm. Subjects demonstrated Snellen 20/800, LogMAR 1.6 acuity with only 25 phosphenes in central vision, improving to Snellen 20/200, LogMAR 1.0 with 375 phosphenes. Comparing results from this study with those from the literature suggests that phosphene patterns mimicking the endogenous center-weighted thalamic map may perform better than patterns with uniform density in poor viewing conditions. Subjects displayed a significant, broad-based improvement in hit rate and response time, suggesting that learning effects were present even in the brief experience from participation in that part of the study. Response time observations implied different strategies might have been employed with different combinations of font size and pattern resolution, suggesting that direct perception might require many more phosphenes than pattern matching although the latter may be sufficient for everyday tasks and slow reading. As learning effects were stronger in the Mid-Range regime, we postulate that future post-implant training should emphasize pattern matching techniques. These findings show that with currently available technology, performance approaching the legal thresholds for blindness is plausible and thus this study has important implications for specifying prosthesis device characteristics such as minimum acceptable contact count and preferred phosphene distribution. Validation of the multiple-optotype two-alternative forced choice paradigm against the Snellen chart tasks provides confidence in the accuracy of the novel task as an assessment tool when using 75% performance level thresholds, although fidelity limitations of the artificial vision simulation suggest that the acuity values reported for the Primary experiment underestimate performance of a future clinical device.

Finally, we propose that the letter recognition task will provide a solid basis for inter-species and inter-laboratory comparisons and that the presented results may prove valuable for selecting prosthesis designs, developing scene analysis software, and guiding post-implant rehabilitation.
